# Effects of Traditional Chinese Medicine Qinbai Qingfei Concentrated Pellet on Cellular Infectivity of *Mycoplasma pneumoniae*

**DOI:** 10.1155/2014/751349

**Published:** 2014-10-29

**Authors:** Guo-Zheng Jiang, Ting Liu, Ji-Chang Li

**Affiliations:** College of Veterinary Medicine, Northeast Agricultural University, Harbin, Heilongjiang 150030, China

## Abstract

*Aim*. To study the effect and mechanism of traditional Chinese medicine Qinbai Qingfei concentrated pellet (QQCP) against *Mycoplasma pneumoniae* (MP). *Methods*. Rat airway smooth muscle (ASM) cells were used to examine the antimycoplasmal activity of QQCP via four drug-adding modes: pre- and postadding drugs, simultaneous-adding after drug and MP mixed, and simultaneous-adding drug and MP; taking roxithromycin dispersive tablets (RDT) as positive control, the cellular *A*_570_ values were determined by MTT method. *Results*. All of *A*_570_ values in QQCP group were significantly higher than those of the corresponding MP control group (*P* < 0.01) in four drug-adding modes; there was no significant difference in *A*_570_ values between the QQCP group and that of the positive control group (*P* > 0.05), confirming that QQCP could significantly inhibit the infectivity of MP to ASM cells. *Conclusion*. QQCP had significant activity in preventing and treating MP infection, killing MP, and antiabsorption.

## 1. Introduction

*Mycoplasma pneumoniae *is a member of the family Mycoplasmataceae and order Mycoplasmatales [1], the smallest self-replicating prokaryotes in both cellular dimensions and genome size [2–4]. Among human* mycoplasmas*, MP is an important pathogen in acute respiratory illnesses in children, accounting for as many as 20% of all cases of community-acquired pneumonia every year [5, 6]. Infections generally lead to tracheobronchitis, bronchiolitis, and interstitial pneumonitis; however 10–15% of such infections progress to primary atypical pneumonia. Recently, studies have shown that adherence of MP to respiratory tract by a terminal structure plays an important role in MP infection. This structure is a membrane-bound protein structure that plays a key role in adhesion, gliding motility movement, and cell division [7, 8]. The lack of cell wall renders* mycoplasmas* insensitive to *β*-lactam antimicrobial agents. They are susceptible to antimicrobial agents that affect DNA, RNA, and protein synthesis. Macrolides and tetracyclines are the most active antibiotics against* mycoplasma*. However, tetracyclines are not approved for use in children younger than 8 years of age due to toxicity to developing cartilage [9]. Recently, the use of new macrolides and fluoroquinolones such as roxithromycin, azithromycin, levofloxacin, and moxifloxacin with more favorable pharmacokinetic property, greater tolerability, and higher level of in vitro activity has been proposed for treatment of* mycoplasma* infections [10]. Unfortunately, studies have shown that treatment with antimicrobials can cause adverse consequences, such as toxicity, side effects, and drug resistance, which have been associated with clinical treatment failure [11, 12]. Recently, a study from Shanghai, China, has reported that 39 of 50 MP isolates tested were macrolide resistant [13], indicating macrolide's limited use. Thus, there is a need to look for a novel clinical therapeutic that not only can against MP, but also can protect respiratory tract.

Qinbai Qingfei concentrated pellet (QQCP) is a traditional Chinese medicine compound preparation developed by Research Institute of Traditional Chinese Medicine of Heilongjiang Province according to clinical experience, used to treat* Mycoplasma* pneumonia for many years in China. The initial results of QQCP in treating* Mycoplasma* pneumonia showed that this drug could effectively alleviate its symptoms [14]. In this study, we authenticate protective effect of QQCP on airway smooth muscle cells from damage by MP.

## 2. Materials and Methods

### 2.1. Preparation of Drug

QQCP was provided kindly by Research Institute of Traditional Chinese Medicine of Heilongjiang Province. It comprises six drugs:* Scutellaria baicalensis* Georgi (major chemical is baicalin),* Pheretima vulgaris* Chen (hypoxanthine is the major active ingredient),* Stemona tuberosa* Lour. (major constituents are tuberostemonine, stemonine, and croomine),* Aster tataricus* L. f. (major active ingredient is butyl-D-ribuloside),* Ophiopogon japonicus* (L. f.) Ker-Gawl. (major constituents are ophiopogonins), and* Platycodon grandiflorus* (Jacq.) A. DC. (the herb contains platycodins). We used HPLC as QQCP standard control marker.

**Figure 1:**
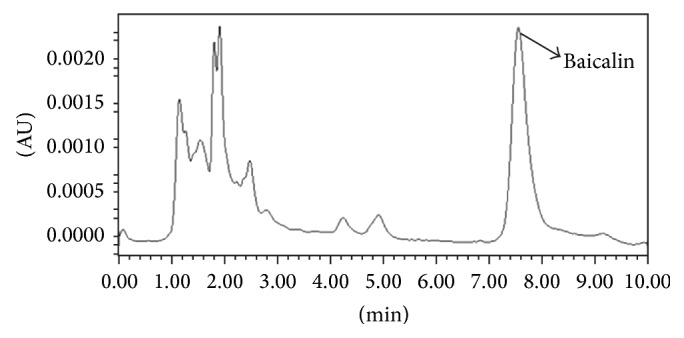
HPLC of “QQCP” preparation extract.

The HPLC system consisted of a LC-10AS pump system (Shimadzu Co., Tokyo, Japan) equipped with a Shimadzu SPD-M10Avp detector, a Shimadzu SCL-10Avp controller, and an SIL-10ADvp autoinjector. The mobile phase was composed of methanol : water : glacial acetic acid (50 : 49 : 1). A Diamonsil-C_18_ column (4.6 mm × 250 mm, 5 *μ*m, DIKMA, Beijing, China) was used. The flow rate was 1.0 mL/min with UV absorbance detection of 270 nm. The analysis involved 10 *μ*L of sample solution. The operating temperature was room temperature (Figure 1).

The QQCP and RDT (Harbin Sixth Pharmaceutical Factory, China) were grinded and diluted into 2% and 0.3% (50 mg crude drug/mL and 3 mg/mL) with deionized water, filtered through a 0.22 *μ*m Millipore membrane filter, and stored at 4°C. For test, they were diluted into eight working concentrations (25–0.195 mg/mL, 1.5–0.012 mg/mL) in twofold serial dilutions with MM containing 5% fetal bovine serum.

### 2.2. *Mycoplasma pneumoniae*

*Mycoplasma pneumoniae* (ATCC15531) was provided kindly by Capital Pediatric Institute of Beijing. MPstrain was grown at 37°C in PPLO broth supplemented with yeast extract, glucose, penicillin, and 20% fetal calf serum and harvested at the turning point from red to yellow as indicated by the indicator system glucose-phenol red. TCID_50_ of the MP liquid was 1 × 10^−2.6^ by Reed-Mueeh assay.

### 2.3. Reagents

PPLO broth was purchased from Hope (Qingdao, China). Dulbecco's modified Eagle's medium (DMEM) (Gibco, USA) supplemented with 10% fetal bovine serum (FBS) was used as growth medium for culturing the cells; for maintenance medium (MM), the serum concentration was reduced to 5% and used for maintaining the cells. Hank's solution was used for washing the trachealis tissue and cells. Trypsin (Gibco, USA) was dissolved with calcium and magnesium-free phosphate-buffered saline (CMF-PBS, pH 7.4) into the concentration of 0.125%. MTT (Gibco, USA), 3-(4,5-dimethylthiazol-2-yl)-2,5-diphenyltetrazolium bromide, was dissolved with CMF-PBS (pH 7.4) into 5 mg mL^−1^. These reagents were filtered through a 0.22 *μ*m Millipore membrane filter. DMEM and MM were stored at 4°C, trypsin solution at −20°C, and MTT solution at 4°C in dark bottles. Dimethyl sulfoxide (DMSO) was purchased from Chemical Agent Company of Chinese Medicine Groups (Nanjing, China). Other chemicals used in the experiment were of analytical grade.

### 2.4. Animals

Male Wistar rats (body weight: 250–300 g) were purchased from Experimental Animal Center of Veterinary Research Institute of Harbin (Harbin, China). All rats (four rats per cage) were fed with water and food* ad libitum* and were kept in the room at the controlled temperature (22 ± 1°C), humidity (50–70%), and 12 h light/dark cycle in the Animal Center of the Northeast Agricultural University. Animal welfare and experimental procedures were carried out in accordance with the guide for the care and use of laboratory animals (National Research Council of USA, 1996) and related ethical regulations of our university.

### 2.5. Methods

#### 2.5.1. Culture of Rat ASM Cells

Primary cultures of rat ASM cells were prepared as described previously with modification [15]. In brief, male Wistar rat weighing 300 g was sacrificed under ether anesthesia and the trachea was excised. The trachea was opened longitudinally and the epithelium was removed by gentle rubbing with a sterile cotton-wool probe. The trachealis muscle was dissected free from the cartilage, washed twice in Hank's solution containing penicillin 100 IU mL^−1^, and then minced with scalpel blades into ~1 mm^2^ pieces. Muscle fragments were placed in culture dishes containing DMEM supplemented with 10% FBS in humidified air containing 5% CO_2_ at 37°C. The culture medium was changed one week later and twice per week thereafter. Upon reaching confluence, cells were passaged by dissociation with 0.125% trypsin and 0.02% EDTA, centrifugation at 1500 rpm for 5 min, and resuspension in culture medium. ASM cells were identified by cytomorphology.

#### 2.5.2. Detection of MP

ASM cells at concentration of 2 × 10^5^ CFU mL^−1^ were seeded in 96-well plates (100 *μ*L/hole) at 37°C in a humid atmosphere of 5% CO_2_. When ASM cells grew into monolayer after cultivation for about 24 h, 100 TCID_50_ MP solution was added to the ASM plate, 100 *μ*L per well. After incubation of 24 h at 37°C in 5% CO_2_, cell supernatant and the third time cell-flushing fluid were centrifuged at 120 00 rpm for 20 min and the supernatant was removed; then 20 *μ*L STET (100 mM NaCl, 10 mM Tris-HCL pH 8.0, 1 mM EDTA pH 8.0, and 5% Triton X-100) was added to DNA extraction. The DNA was subjected to PCR.

According to MP sequences described by Bernet et al. [16], the primers for detection of MP (MP-1: 5′-TTAGCAGGTAATGGCTAGAG-3′; MP-2: 5′-CTCGGTTAACCTCAATTATG-3′) were designed by Primer Premier 5.0. The PCR mixture included 2.8 *μ*L of 10 × PCR buffer, 200 mmol L^−1^ of dNTPs, 0.5 *μ*mol L^−1^ of each primer, 1 U of Taq DNA polymerase, and 8 *μ*L of DNA solution. Amplification conditions were pre-denaturation at 95°C for 5 min, followed by denature at 95°C for 40 s, annealing at 55°C for 1 min, extension at 72°C for 1 min, cycling 35 times, and last extension for 10 min. PCR was performed in a S1000TM Thermal Cycler (Bio-RAD, NY, USA). For each PCR pure extracted DNA from MP (ATCC15531) was used as positive control. PCR products were analyzed by agarose gel electrophoresis (2% agarose) and ethidium bromide staining.

#### 2.5.3. Cytotoxicity Analysis

The safe concentrations of two drugs for ASM cells were measured by the cytopathic effect (CPE) method. QQCP was diluted twofold serially from 50 mg mL^−1^ into 8 concentrations with MM, RDT from 3 mg mL^−1^. ASM cells were prepared and diluted into 2 × 10^5^ CFU mL^−1^ with MM and inoculated into 96-well plates (100 *μ*L/hole) at 37°C in a humid atmosphere of 5% CO_2_ for use.

When ASM cells grew into monolayer after cultivation for about 24 h, in two groups, QQCP and RDT at series of concentrations were added to the plates, respectively, four wells for each concentration. At the same time, cell control group (only adding MM) and blank group (no cell) were designed. After cultivation for 72 h at 37°C in a humid atmosphere of 5% CO_2_, cells were observed under inverted microscope, the highest concentration of drug did not display cellular shrinkage, and detachment compared with cell control group was the largest safety concentration of drug.

#### 2.5.4. Protection of QQCP on Airway Smooth Muscle Cells

According to the result of cytotoxicity analysis, two drugs at two concentrations, QQCP from 3.125 to 1.563 mg mL^−1^, RDT from 0.047 to 0.024 mg mL^−1^, were used for determination of antimycoplasmal activity by MTT method. When ASM cells were cultured into monolayer, the serial twofold dilutions of drugs and MP solution were added to cell plate, respectively, in four manners.

*Preadding Drug*. Drug solutions were added to ASM cells plate firstly, 100 *μ*L per well and four wells per concentration. After incubation for 24 h at 37°C in 5% CO_2_, the drug solutions were removed, the cell was washed twice with Hank's solution, and the MP solution was added. 

*Postadding Drug*. MP solution was added to ASM cells plate firstly, 100 *μ*L per well. After incubation of 2 h at 37°C in 5% CO_2_, the MP solution was removed, the cell was washed twice with Hank's solution, and the drug solutions were added, four wells for each concentration. 

*Simultaneous-Adding after Drug and MP Mixed*. The drug solutions at each concentration were mixed with MP solution, incubated for 2 h at 37°C in 5% CO_2_, and then centrifuged at 50 000 rpm for 1 h and the supernatant was removed. The MP was washed twice, and then the MP solution without drug was added to ASM cells plate, 100 *μ*L per well and four wells per concentration. 

*Simultaneous-Adding Drug and MP*. MP solution was added to ASM cells plate, then drug solutions were added immediately, 100 *μ*L per well and four wells per concentration. After incubation for 1 h at 4°C, the supernatant was removed and the MM solution was added.

At the same time, the cell control group (only adding MM), the MP control group (only adding MP), and blank group (no cell) were designed. All the plates were placed in 5% CO_2_ incubator at 37°C. When the cells displayed cellular shrinkage, and detachment in MP control group, 20 *μ*L MTT was added to each well and sequentially incubated for 4 h, the supernatant was removed, and 100 *μ*L of DMSO was added to each well to dissolve the purple formazan crystals. The plates were shaken for another 5 min. Finally, the absorbance at 570 nm (*A*_570_ value) of each well was measured by microliter enzyme-linked immunosorbent assay reader (Bio-RAD, NY, USA). The MP inhibitory rate was calculated based on the following formula: MP inhibitory rate = (${\overset{-}{A}}_{570}$ value of drug group −  ${\overset{-}{A}}_{570}$ value of MP control)/(${\overset{-}{A}}_{570}$  value of cell control group $-\;\;{\overset{-}{A}}_{570}$ value of MP control) × 100% (the  ${\overset{-}{A}}_{570}$  value was the average of four wells). The *A*_570_ values and MP inhibitory rate were considered as the indicator of antimycoplasmal activity.

### 2.6. Statistical Analysis

Values were expressed as mean ± SD. Differences between groups were determined by analysis of variance (ANOVA) using the Sigma Stat statistical software (SPSS Science, Chicago, USA). A value of *P* < 0.05 was considered statistically significant.

## 3. Results

### 3.1. Morphology of ASM Cells

**Figure 2:**
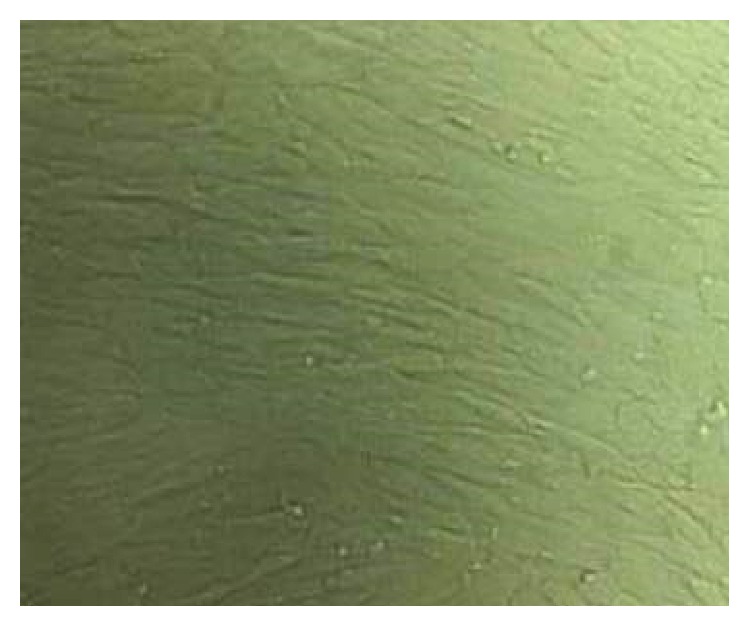
Morphology of airway muscle cells. Rat airway muscle cells grew into monolayer at long shuttle-type, identical shape and size and arranged closely (×40).

After incubation of 3 d, spindle cells appeared around the muscle fragments. On the 7th day, cells reached confluence and displayed the typical “hill and valley” (multilayer and monolayer cells) pattern. The muscle fragments were removed and dissociated. ASM cells grew into monolayer at long shuttle-type, identical shape and size and arranged closely (Figure 2) after cultivation for 3 d.

### 3.2. Detection of MP

**Figure 3:**
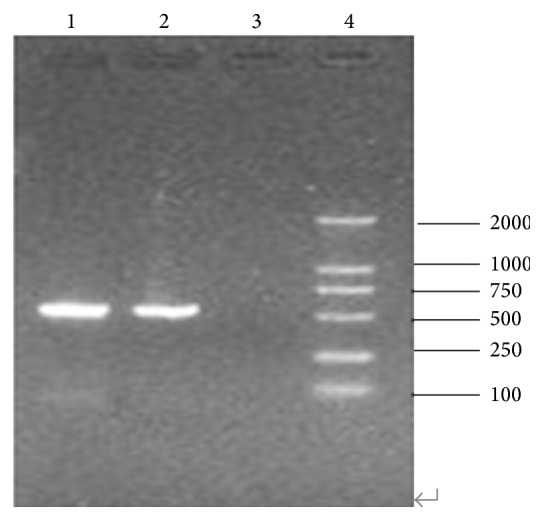
Gel electrophoresis results of PCR products. The third time cell-flushing fluid and supernatant were collected after cultivation for 24 h and subjected to PCR analysis.* Mycoplasma pneumoniae *was detected in supernatant (Lane 2), but not in the cell-flushing fluid (Lane 3). Lane 1 is* Mycoplasma pneumoniae* (ATCC 15531) positive control; Lane 4 is molecular weight standard.

**Figure 4:**
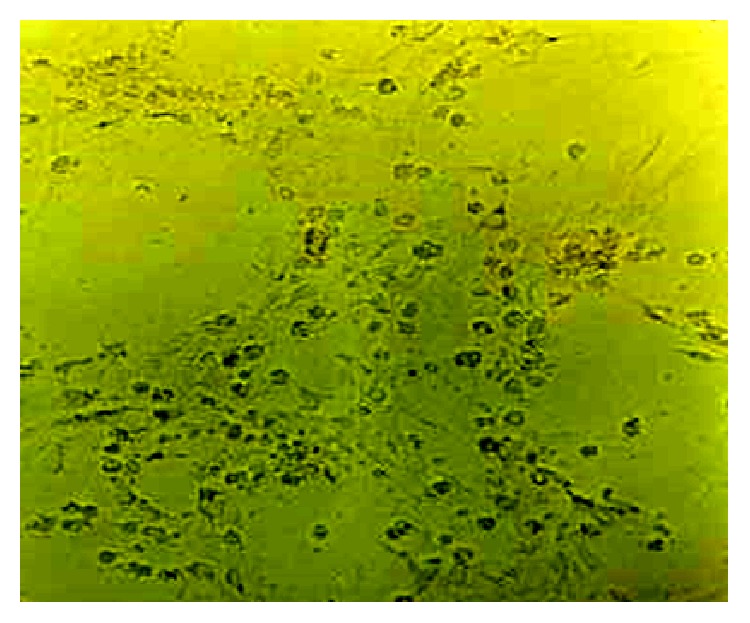
Morphology of airway muscle cells after exposure to* Mycoplasma pneumoniae*. Cells became unclear in the structure, atrophy, round, and shedding after cultivation for 7 d (×40).

The third time cell-flushing fluid and supernatant were collected after cultivation for 24 h and were subject to PCR analysis. MP was detected in supernatant, but not in the cell-flushing fluid (Figure 3). The ASM cells became round firstly, then distinct lesion with some cells collapsing and falling off (Figure 4) was observed after incubation for 7 d with MP; the normal cells with clear cell structure arranged closely and each cell was shuttle-type.

### 3.3. The Cytotoxicity of Drugs

The cells at 6.25 mg mL^−1^ QQCP group and 0.094 mg mL^−1^RDT group did not show collapsing and falling off compared with cell control group. Therefore, these concentrations could be considered as their maximal safety concentrations.

### 3.4. Antimycoplasmal Activity in Preadding Drug

The *A*_570_ values and MP inhibitory rates of every group were listed in Table 1. The *A*_570_ values of QQCP at 3.125 and 1.563 mg mL^−1^ groups were significantly higher than those of the corresponding MP control group (*P* < 0.01). The *A*_570_ values of RDT were not significantly higher than those of the corresponding MP control group (*P* > 0.05). QQCP (44.08%) at 3.125 mg mL^−1^ and RDT (15.59%) at 0.024 mg mL^−1^ group presented the highest MP inhibitory rate.

### 3.5. Antimycoplasmal Activity in Postadding Drug

The *A*_570_ values and MP inhibitory rates of every group were listed in Table 2. The *A*_570_ values of QQCP at 3.125 and 1.563 mg mL^−1^ group and RDT at 0.047 and 0.024 mg mL^−1^ were significantly higher than those of the corresponding MP control group (*P* < 0.01). QQCP (89.36%) at 3.125 mg/mL and RDT (95.80%) at 0.024 mg mL^−1^ group presented the highest MP inhibitory rate.

### 3.6. Antimycoplasmal Activity in Simultaneous-Adding after MP and Drug Mixed

The *A*_570_ values and MP inhibitory rates of every group were listed in Table 3. The *A*_570_ values of QQCP at 3.125 and 1.563 mg mL^−1^ group and RDT at 0.047 and 0.024 mg mL^−1^ were significantly higher than those of the corresponding MP control group (*P* < 0.01). QQCP (86.66%) at 3.125 mg mL^−1^ and RDT (95.50%) at 0.047 mg mL^−1^ group presented the highest MP inhibitory rate.

### 3.7. Antimycoplasmal Activity in Simultaneous-Adding MP and Drug

The *A*_570_ values and MP inhibitory rates of every group were listed in Table 4. The *A*_570_ values of QQCP at 3.125 and 1.563 mg mL^−1^ group and RDT at 0.047 and 0.024 mg mL^−1^ were significantly higher than those of the corresponding MP control group (*P* < 0.01). QQCP (55.02%) at 3.125 mg mL^−1^ and RDT (85.91%) at 0.047 mg mL^−1^ group presented the highest MP inhibitory rate.

## 4. Discussion

To date, the pathogenesis of* Mycoplasma* pneumonia has not clear yet; the main pathogenesis tends to be adhesion and penetration of MP, direct injury to cells, and inflammation reaction [10]. Adhesion of MP to host cells is a prerequisite for colonization by the parasite and for infection [17]. This close interaction between the MP and host cells protects it from removal by the host's mucociliary clearance mechanism and allows it to produce local cytotoxin effects. Because MP depends on close association with host cells to survive, it has evolved a complex and specialized attachment organelle to facilitate its parasitic existence. This attachment organelle consists of a specialized tip structure with a central core of a dense rodlike central filament surrounded by a lucent space that is enveloped by an extension of the organism's cell membrane. The tip structure is actually a network of adhesins, interactive proteins, and adherence accessory proteins that cooperate structurally and functionally to mobilize and concentrate adhesins at the tip of organism [18, 19]. The P1 adhesin is a 169-kDa protein concentrated in the attachment tip that is now known to be the major structure responsible for interaction of MP with host cells [8, 20]. In addition to P1, P30, P116, and HMW1-3 are believed to participate in the establishment of the polar structure [7, 21, 22].

In the present experiment, 100 TCID_50_ MP solution was added to the ASM cells plate. The third time cell-flushing fluid and supernatant were collected in the following day and were subject to PCR analysis. MP was detected in supernatant, but not in the cell-flushing fluid. After cultivation for 7 d, cells became unclear in the structure, atrophy, round, and shedding, even scattered at end. These results showed that MP had strong adherence to ASM cells, causing significant damages to the respiratory tract. These damages may be caused by penetration of MP, direct injury to cells.

This experiment in antimycoplasmal activity designed four drug-adding modes [23]. The preadding drug mode is thought to exert the inhibitory action at a very early stage of MP infection cycle, that is, MP adsorption or penetration into the host cell. In agreement with this idea, our results showed that all only the *A*_570_ values of QQCP at 2 concentration groups were significantly higher than those of the corresponding MP control group, which indicated that QQCP could prevent MP infection. This effect may be due to blockade of drug in MP binding to cell receptor during adsorption, the first step of infection. The postadding drug mode is thought to exert the disturb action from MP metabolism. It may affect the transmembrane and intracellular signaling processes involved in regulating MP expression. In agreement with this idea, our results showed that all *A*_570_ values of two drug groups at 2 concentration groups were significantly higher than those of the corresponding MP control group, which indicated that they could treat MP infection. The simultaneous-adding after MP and drug mixed is thought to be the direct inactivation of MP. Our results showed that all *A*_570_ values of two drug groups at 2 concentration groups were significantly higher than those of the corresponding MP control group. The simultaneous-adding MP and drug mode is thought to prevent sticking of MP to the host cell. It may affect the protein of tip structure, which plays a key role in adhesion. Previous study [24] showed that QQCP can inhibit P1 expression. Our results showed that all *A*_570_ values of two drug groups at 2 concentration groups were significantly higher than those of the corresponding MP control group. These results confirmed that both QQCP and RDT had significant activity in killing MP, treating MP infection, and antiabsorption. At the same time, QQCP could prevent MP infection, while RDT did not possess this action.

## Figures and Tables

**Table 1:** The *A*_570_ values and MP inhibitory rates of every group in preadding drug (*n* = 4).

Concentration (mg/mL)	QQCP		Concentration (mg/mL)	RDT	
	*A*_ 570_	Inhibitory rate (%)		*A*_ 570_	Inhibitory rate (%)
3.125	0.679 ± 0.286^*^	44.08	0.047	0.442 ± 0.036	8.55
1.563	0.593 ± 0.189^*^	31.1	0.024	0.489 ± 0.130	15.59
MP	0.385 ± 0.117	—	MP	0.385 ± 0.117	—
Cell	1.052 ± 0.176	—	Cell	1.052 ± 0.176	—

Note: QQCP: Qinbai Qingfei concentrated pellet group; RDT: roxithromycin dispersive tablets group; MP: *Mycoplasma pneumoniae* control group; cell: cell control group. Values are expressed as the mean ± SD.

^*^ *P* < 0.01 compared with MP control group.

**Table 2:** The *A*_570_ values and MP inhibitory rates of every group in postadding drug (*n* = 4).

Concentration (mg/mL)	QQCP		Concentration (mg/mL)	RDT	
	*A*_ 570_	Inhibitory rate (%)		*A*_ 570_	Inhibitory rate (%)
3.125	0.981 ± 0.192^*^	89.36	0.047	1.013 ± 0.196^*^	94.15
1.563	0.964 ± 0.205^*^	86.81	0.024	1.024 ± 0.450^*^	95.80
MP	0.385 ± 0.117	—	MP	0.385 ± 0.117	—
Cell	1.052 ± 0.176	—	Cell	1.052 ± 0.176	—

Note: QQCP: Qinbai Qingfei concentrated pellet group; RDT: roxithromycin dispersive tablets group; MP control group: *Mycoplasma pneumoniae* control group; cell: cell control group. Values are expressed as the mean ± SD.

^*^ *P* < 0.01 compared with MP control group.

**Table 3:** The *A*_570_ values and MP inhibitory rates of every group in simultaneous-adding after MP and drug mixed (*n* = 4).

Concentration (mg/mL)	QQCP		Concentration (mg/mL)	RDT	
	*A*_ 570_	Inhibitory rate (%)		*A*_ 570_	Inhibitory rate (%)
3.125	0.963 ± 0.061^*^	86.66	0.047	1.022 ± 0.256^*^	95.50
1.563	0.952 ± 0.106^*^	85.50	0.024	1.013 ± 0.352^*^	94.15
MP	0.385 ± 0.117	—	MP	0.385 ± 0.117	—
Cell	1.052 ± 0.176	—	Cell	1.052 ± 0.176	—

Note: QQCP: Qinbai Qingfei concentrated pellet group; RDT: roxithromycin dispersive tablets group; MP control group: *Mycoplasma pneumoniae* control group; cell: cell control group. Values are expressed as the mean ± SD.

^*^ *P* < 0.01 compared with MP control group.

**Table 4:** The *A*_570_ values and MP inhibitory rates of every group in simultaneous-adding MP and drug (*n* = 4).

Concentration (mg/mL)	QQCP		Concentration (mg/mL)	RDT	
	*A*_ 570_	Inhibitory rate (%)		*A*_ 570_	Inhibitory rate (%)
3.125	0.752 ± 0.261^*^	55.02	0.047	0.958 ± 0.306^*^	85.91
1.563	0.738 ± 0.105^*^	52.92	0.024	0.932 ± 0.106^*^	82.01
MP	0.385 ± 0.117	—	MP	0.385 ± 0.117	—
Cell	1.052 ± 0.176	—	Cell	1.052 ± 0.176	—

Note: QQCP: Qinbai Qingfei concentrated pellet group; RDT: roxithromycin dispersive tablets group; MP control group: *Mycoplasma pneumoniae* control group; cell: cell control group. Values are expressed as the mean ± SD.

^*^ *P* < 0.01 compared with MP control group.

## Abbreviations

QQCP: Qinbai Qingfei concentrated pellet
MP: * Mycoplasma pneumoniae*
ASM: Airway smooth muscle
RDT: Roxithromycin dispersive tablets
DMEM: Dulbecco's modified Eagle's medium
FBS: Fetal bovine serum
MM: Maintenance medium
MTT: 3-(4,5-Dimethylthiazol-2-yl)-2,5-diphenyltetrazolium bromide
DMSO: Dimethyl sulfoxide
CMF-PBS: Calcium and magnesium-free phosphate-buffered saline
EDTA: Ethylene diamine tetraacetic acid
CPE: Cytopathic effect
TCID_50_: Tissue culture infectious dose 50
PCR: Polymerase chain reaction.
